# Supplementary material for: Segmented School Physical Activity and Weight Status in Children: Application of Compositional Data Analysis
Source: Int J Environ Res Public Health. 2021 Mar 21;18(6):3243. doi: 10.3390/ijerph18063243 (PMC8003978; doi:10.3390/ijerph18063243)
Supplement: Supplementary file 1 [file ijerph-18-03243-s001.pdf]

Methods for Compositional Data Analyses (CoDA)

The following CoDA procedures were adopted from prior published work that used accelerometer physical activity time use data. Steps per school day during physical education (PE), recess (RECESS,) lunchtime (LUNCH), and the academic class time (CLASS) were converted to percentage of total school day step activity within each segment so that their sum was equal to 100%. The geometric means were calculated for each school segment. The geometric compositional value represents percentage of total school day step activity based on a 7-h school day.

Compositional data occupy a quotient space which can be represented in a D-part simplex with four compositional parts. However, in order to analyze the data in real space, log-ratio data transformations were performed. Isometric Log Ratio coordinates (*ilr*s) were calculated using the following equations:

$$ilr1 = \sqrt{\frac{3}{4}}\ \ln\left(\frac{\text{PE}}{(\text{RECESS} \times \text{LUNCH} \times \text{CLASS})^{\frac{1}{3}}}\right) \quad (1)$$

$$ilr2 = \sqrt{\frac{2}{3}}\ \ln\left(\frac{\text{RECESS}}{(\text{LUNCH} \times \text{CLASS})^{\frac{1}{2}}}\right) \quad (2)$$

$$ilr3 = \sqrt{\frac{1}{2}}\ \ln\left(\frac{\text{LUNCH}}{\text{CLASS}}\right) \quad (3)$$

Here, *ilr*1 expresses the ratio of percentage of school step activity during PE to percentage of school step activity during all other non-PE school day segments. The *ilr*2 is the ratio of percentage of school steps during RECESS in relation to LUNCH and CLASS. Finally, *ilr*3 is the ratio of percentage of school day steps during LUNCH in relation to % of steps accrued during CLASS. These three *ilr*s were included in the linear regression analyses; however, the inferences about the primary contrast of interest (i.e., PE relative to the three non-PE school segments) was based *ilr*1. Because of this, additional *ilr*s were calculated by permutating school segments in a sequential manner to obtain interpretable parameter estimates for the other segments, specifically: RECESS (*ilr*s 4-6), LUNCH (*ilr*s 7-9) and CLASS (*ilr*s 10-12).

$$ilr4 = \sqrt{\frac{3}{4}}\ \ln\left(\frac{\text{RECESS}}{(\text{PE} \times \text{LUNCH} \times \text{CLASS})^{\frac{1}{3}}}\right) \tag{4}$$

$$ilr5 = \sqrt{\frac{2}{3}}\ \ln\left(\frac{\text{PE}}{(\text{LUNCH} \times \text{CLASS})^{\frac{1}{2}}}\right) \tag{5}$$

$$ilr6 = \sqrt{\frac{1}{2}}\ \ln\left(\frac{\text{LUNCH}}{\text{CLASS}}\right) \tag{6}$$

$$ilr7 = \sqrt{\frac{3}{4}}\ \ln\left(\frac{\text{LUNCH}}{(\text{PE} \times \text{RECESS} \times \text{CLASS})^{\frac{1}{3}}}\right) \tag{7}$$

$$ilr8 = \sqrt{\frac{2}{3}}\ \ln\left(\frac{\text{PE}}{(\text{RECESS} \times \text{CLASS})^{\frac{1}{2}}}\right) \tag{8}$$

$$ilr9 = \sqrt{\frac{1}{2}}\ \ln\left(\frac{\text{RECESS}}{\text{CLASS}}\right) \tag{9}$$

$$ilr10 = \sqrt{\frac{3}{4}}\ \ln\left(\frac{\text{CLASS}}{(\text{PE} \times \text{RECESS} \times \text{LUNCH})^{\frac{1}{3}}}\right) \tag{10}$$

$$ilr11 = \sqrt{\frac{2}{3}}\ \ln\left(\frac{\text{PE}}{(\text{RECESS} \times \text{LUNCH})^{\frac{1}{2}}}\right) \tag{11}$$

$$ilr12 = \sqrt{\frac{1}{2}}\ \ln\left(\frac{\text{RECESS}}{\text{LUNCH}}\right) \tag{12}$$

Therefore, the *ilr*s from Equations (1)-(3) were entered into each linear model to obtain the parameter estimate for $ilr_{\text{PE/RECESS×LUNCH×CLASS}}$, the *ilr*s from Equations (4)-(6) were entered into each linear model to obtain the parameter estimate for $ilr_{\text{RECESS/PE×LUNCH×CLASS}}$, the *ilr*s from Equations (7)-(9) were entered into each linear model to obtain the parameter estimate for $ilr_{\text{LUNCH/ PE×RECESS×CLASS}}$, and the *ilr*s from Equations (10)-(12) were entered into each linear model to obtain the parameter estimate for $ilr_{\text{CLASS/ PE×RECESS×LUNCH}}$. Because of the permutation principle, each respective linear model with four school day segments (PE, RECESS, LUNCH, CLASS) will have the same estimated fit, intercept, and *p*-value per permutation.

Table S1. Arithmetic and geometric compositional step count means and %'s for each school day segment (based on a 7-h school day).

	Arithmetic Mean		Compositional Mean	
	Steps/day	% of Total School Day Steps	Steps/day	% of Total School Day Steps
PE	1600	29.3%	1482	31.1%
RECESS	1071	19.6%	978	20.5%
LUNCH	1508	27.6%	1418	29.8%
CLASSROOM	1276	23.4%	888	18.6%

Note: PE is step counts during physical education; RECESS is step counts during recess; LUNCH is step counts during lunch period; CLASS is step counts within the academic classroom.

Table S2. Compositional variation matrix.

	PE	RECESS	LUNCH	CLASS
PE	-			
RECESS	0.40	-		
LUNCH	0.18	0.14	-	
CLASS	1.39	1.23	1.12	-

Note: PE is step counts during physical education; RECESS is step counts during recess; LUNCH is step counts during lunch period; CLASS is step counts within the academic classroom; Lower cell values indicate greater co-dependence between school segment steps; higher cell values indicate greater independence between school segment steps.

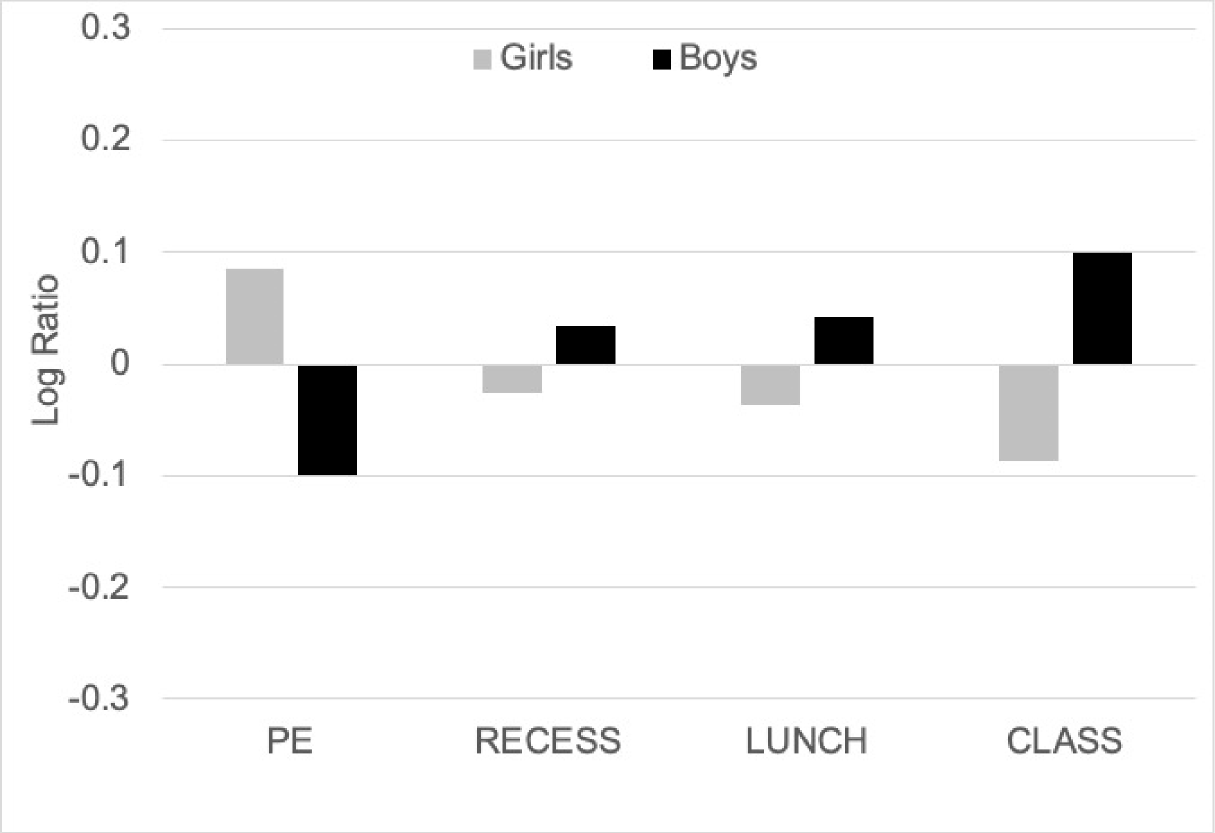

Figure S1. Geometric mean bar plot showing the log ratio of % of total school day step counts accrued within physical education, recess, lunch, and within the academic classroom, stratified by sex.

Note: Each bar represents the geometric mean of the specific group (g_k), expressed as a ratio measured on a logarithmic scale to the geometric mean of the entire sample ($\ln g_k / g$). A ratio of 0 reflects that the geometric means of the specific group and the entire sample are equal. Positive and negative values show that the group geometric mean is larger and smaller than the entire sample, respectively. PE is step counts during physical education; RECESS is step counts during recess; LUNCH is step counts during lunch period; CLASS is step counts within the academic classroom.
